# Improving Structural Homogeneity, Hydraulic Permeability, and Mechanical Performance of Asymmetric Monophasic Cellulose Acetate/Silica Membranes: Spinodal Decomposition Mix

**DOI:** 10.3390/membranes13030346

**Published:** 2023-03-17

**Authors:** Fahimeh Zare, Sérgio B. Gonçalves, Mónica Faria, Maria Clara Gonçalves

**Affiliations:** 1 Departamento de Engenharia Química, Instituto Superior Técnico, Universidade de Lisboa, 1049-001 Lisboa, Portugal; 2 Centro de Química Estrutural (CQE), 1049-001 Lisboa, Portugal; 3 IDMEC, Instituto de Engenharia Mecânica, Instituto Superior Técnico, Universidade de Lisboa, 1049-001 Lisboa, Portugal; 4 Center of Physics and Engineering of Advanced Materials (CeFEMA), Laboratory for Physics of Materials and Emerging Technologies (LaPMET), Chemical Engineering Department, Instituto Superior Técnico, Universidade de Lisboa, 1049-001 Lisbon, Portugal

**Keywords:** monophasic hybrid membrane, CA/SiO_2_ membrane, spinodal decompositions mix, integral asymmetric membranes, hydraulic permeability, mechanical tensile properties

## Abstract

In this paper, we propose an optimized protocol to synthesize reproducible, accurate, sustainable integrally skinned monophasic hybrid cellulose acetate/silica membranes for ultrafiltration. Eight different membrane compositions were studied, divided into two series, one and two, each composed of four membranes. The amount of silica increased from 0 wt.% up to 30 wt.% (with increments of 10 wt.%) in each series, while the solvent composition was kept constant within each series (formamide/acetone ratio equals 0.57 wt.% in series one and 0.73 wt.% in series two). The morphology of the membranes was analyzed by scanning electron microscopy and the chemical composition by Fourier transform infrared spectroscopy, in attenuated total reflection mode (FTIR-ATR). Mechanical tensile properties were determined using tensile tests, and a retest trial was performed to assess mechanical properties variability over different membrane batches. The hydraulic permeability of the membranes was evaluated by measuring pure water fluxes following membrane compaction. The membranes in series two produced with a higher formamide/acetone solvent ratio led to thicker membranes with higher hydraulic permeability values (47.2–26.39 kg·h^−1^·m^−2^·bar^−1^) than for the membranes in series one (40.01–19.4 kg·h^−1^·m^−2^·bar^−1^). Results obtained from the FTIR-ATR spectra suggest the presence of micro/nano-silica clusters in the hybrid membranes of series one, also exhibiting higher Young’s modulus values than the hybrid membranes in series two.

## 1. Introduction

Polymeric membranes account for the largest market share in membrane filtration technology, particularly in terms of water purification and the desalination processes [1,2]. Likewise, the emerging artificial organ technology focuses on the design and development of novel hemocompatible polymeric membranes to assist the physical and chemical functions of failed organs, such as lungs and kidneys [3,4,5,6]. Cellulose acetate (CA), a natural biodegradable thermoplastic polymer derived from a low-cost and renewable source, stands out for its large availability and respect for the environment. Other characteristics, such as high mechanical flexibility, excellent water affinity, low protein adsorption, ease of processing, and good film-forming properties, render CA a pivotal role in the design of sustainable polymeric membranes. When pharmaceutical and medical applications are envisaged, and the biocompatible functionalization of membranes is needed, monophasic hybrid CA/silica (SiO_2_)-based membranes emerge as a promising route. Furthermore, these hybrid CA/SiO_2_ membranes may overcome some of the weaknesses attributed to pristine CA membranes by mitigating likely fouling [7] and enhancing mechanical strength [8].

Gonçalves and Faria pioneered an innovative strategy to fabricate monophasic hybrid CA/SiO_2_-based membranes [7,8,9,10,11,12] by combining the membranes’ phase inversion technique [13] with sol–gel methodology [14,15]. In this approach, in situ homo- and hetero-condensation reactions take place during casting solution homogenization (under acid catalysis) between silanols (≡Si–OH−) from the hydrolyzed silica precursors and silanols and hydroxyls (OH−) from the CA polymer, respectively. Detailed FTIR-ATR characterization of the chemical composition of the monophasic hybrid CA/SiO_2_-based membranes revealed that SiO_2_ tetrahedra are covalently bound to the organic matrix and relatively well distributed throughout the polymer network [8,11,16].

Despite the encouraging results of CA/SiO_2_-based membranes in terms of mechanical properties and permeation performance, the low reproducibility of the membrane properties, such as active layer-thickness and mechanical and permeation performance, need to be outstripped. A better understanding of the formation of the membranes during co-polymerization and phase separation process becomes critical to develop reproducible accurate fabrication protocols. To help figure out the monophasic hybrid CA/SiO_2_ membranes formation process, polymer-solvent–nonsolvent spinodal decomposition along with hybrid casting solution reactional processes are revisited now.

### 1.1. Polymer-Solvent-Nonsolvent Spinodal Decomposition

**Figure 1:**
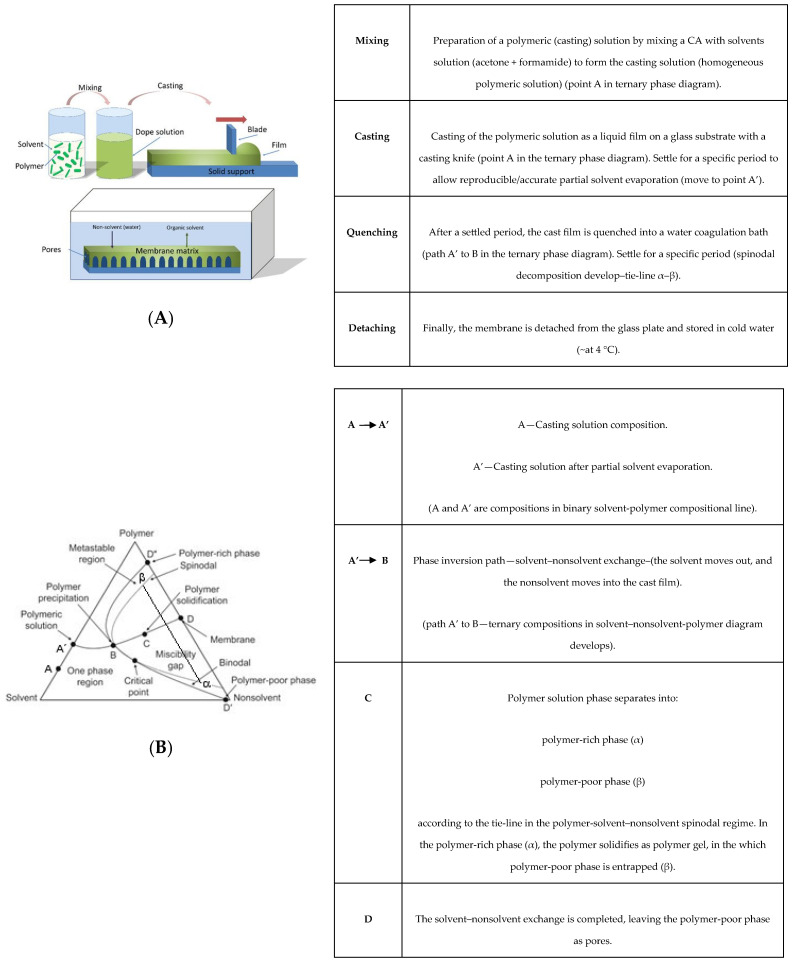
Polymer-solvent–nonsolvent system: (**A**). Classical Loeb–Sourirajan method setup; (**B**). Ternary phase diagram.

Pristine CA membranes are commonly produced by the classical Loeb–Sourirajan technique [13], a nonsolvent induced phase separation method, comprising the sequential steps: mixing, casting, quenching, and detaching (illustrated in Figure 1). In the present work, CA is the polymer, acetone-formamide the solvent mixture, and water the nonsolvent compound.

By mixing CA polymer with a solvent mixture (acetone and formamide, in adequate proportions), a homogeneous polymeric solution forms (the casting solution) (point A in the binary compositional line polymer-solvent, Figure 1B).

The polymeric solution (A) is then cast onto a subtract and then settled for a set period (to allow reproducible/accurate partial solvent evaporation). During this time, the more volatile solvent (acetone in the present case) partially evaporates, enriching the casting film in the polymer phase (as point A moves towards A′, along the binary line polymer-solvent). An asymmetric polymer membrane (with top skin layer) forms.

The membrane is sequentially quenched in water (nonsolvent bath). The solvent–nonsolvent exchange takes place. Formamide (the less volatile solvent) and water (nonsolvent) are the players (both polar protic molecules, Figure 1B). The quenched polymeric film composition leaves the binary compositional line (polymer-solvent edge on the ternary diagram, Figure 1B) and follows A′-B path (in the ternary domain).

At some point, the solvent content (formamide and acetone) in the polymeric film becomes so low that it can no longer hold CA polymer (in one phase) and promotes phase separation. If phase separation occurs in the region marked out by binodal and spinodal lines (a thermodynamic metastable region), the solid polymer precipitates out of the liquid through nucleation and growth mechanisms. If the spinodal line is overtripped, a thermodynamically unstable region is reached, and a 3D interconnected spinodal morphology appears between a polymer-rich phase (*α*) and a polymer-poor phase (*β*) (Figure 1B). In the Loeb–Sourirajan phase inversion method, the presence of a spinodal miscibility gap is mandatory. Spinodal phase separation phenomena are common in polymeric solutions and were thermodynamically described by Flory–Huggins theory [17,18,19,20], centered on the expression for the free energy of mixing derived from a lattice model. The theory is constituted by combinatorial entropy terms associated with polymer chain configurations on the lattice, and an enthalpic contribution owing to interactions between the different species. The Gibbs free energy change of mixing, Δ*G_m_*, is given by the following equation:

Δ*G_m_* = Δ*H_m_* − *T*Δ*S_m_* ∝ *RT*(*ϕ*_1_*P*_1_ln*ϕ*_1_ + *ϕ*_2_*P*_2_ln*ϕ*_2_ + χ_12_*ϕ*_1_*ϕ*_2_) (1)

where *H_m_* and *S_m_* represent, respectively, the enthalpy and entropy terms associated with the mixing process, *R* is the gas constant (J·mol^−1^·K^−1^), *T* is the absolute temperature (K), *ϕ_i_* and *P_i_* are the volume fractions and the degrees of polymerization of the two different components (*i* = 1 and *i* = 2) that can be polymerizable (or not, e.g., in the case of solvents), and $\chi_{12}$ is the Flory interaction parameter, which describes the chemical compatibility between both components. The first two terms inside the parenthesis in Equation (1) are related to the entropic contribution to Δ*G_m_*, while the last term is related to the enthalpic contribution of the system [21].

### 1.2. Hybrid Casting Solution Reactional Processes

To produce monophasic hybrid CA/SiO_2_ membranes, a sol–gel silica system composed of water, acid and tetraethoxysilane (TEOS) (the most common silica sol–gel precursor) is introduced in the polymeric casting solution (mixture of CA, acetone and formamide) bringing additional complexity to the phase diagram and the membrane preparation.

**Figure 2:**
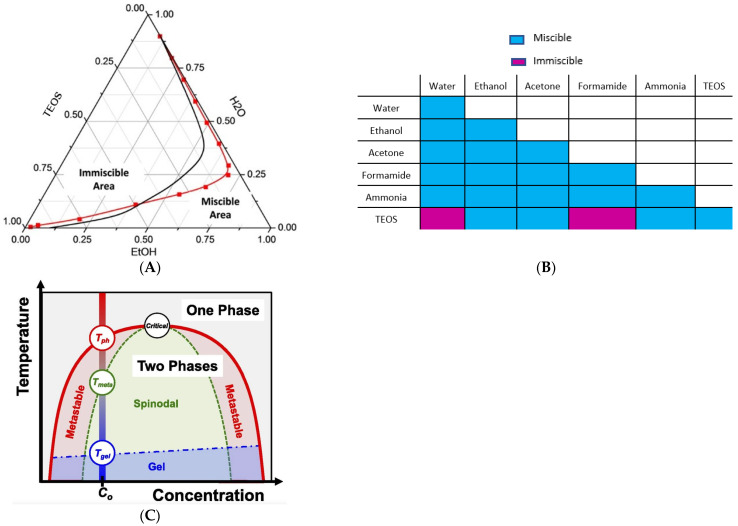
Silica sol–gel system: (**A**) ternary TEOS-EtOH-H_2_O system; (**B**) chemical miscibility/immiscibility between solvents and reactants used in polymer and sol–gel silica systems; (**C**) spinodal phase separation: phase diagram.

Concerning the sol–gel process, as TEOS is immiscible with water (the two reactants to produce silica), a co-solvent (Figure 2A,B) is mandatory, ethanol being the most used. In the present study, ethanol is efficiently replaced by acetone. Acetone (an aprotic polar solvent) favors the $S_{N}2$ acid catalyzed hydrolysis of TEOS over ethanol, as ethanol (being a polar protic solvent) hydrogen bonds with -OH, =O or -O- (present in silica, hydrolyzed silica precursor and acetate molecules), retarding the kinetics of hydrolysis [14].

Condensation (that happens sequentially at low pH values) depends on the number of hydrolyzed species present, so by favoring hydrolysis, acetone favors condensation reactions, processes that develop during casting solution preparation.

Further, the binary system silica-formamide exhibits an additional singularity—a well-studied spinodal decomposition [21,22,23,24] (Figure 2). So, in the present hybrid CA/SiO_2_ casting solution, two spinodal decomposition systems mix–CA-solvent–nonsolvent (Figure 1) and TEOS-formamide (Figure 2). When multiple-phase separation processes are present in a particular system, they progress sequentially. Initial phase separation forms two phases, which then become isolated subsystems within which further immiscibility develops with cooling. Secondary phase separation leads to a microstructure of phases within primary phases [25]. 

The silica sol–gel reactional processes, which take place during casting solution preparation, start with the silica precursor hydrolysis (TEOS in our case):

Si(OCH_2_CH_3_)_4_ + 4 -OH → Si(OH)_4_ + 4 CH_2_CH_2_OH (2)

This is immediately followed by homo- or hetero-polymerization (at low pH values) with either silanol groups (Equation (3)) or -OH from CA polymer (Equation (4)) [8]:

Si(OH)_4_ + Si(OH)_4_ → Si_2_O(OH)_6_ + H_2_O (3)

≡Si-OH + HO-C≡ → Si-O-C + H_2_O (4)

The richness and complexity of the membrane polymerization process are thus highly amplified, resulting in a more complex hybrid network, which inevitably influences the mechanical and hydraulic performances of the membrane. The complexity created by homo- and hetero-condensation reactions decrease the entropy change (Δ*S_m_*) and increase the enthalpy contribution (Δ*H_m_*) of the system due to the arising of polarity differences between the new molecular chains and solvent molecules (increasing $\chi_{12}$) [21]. Both of these effects contribute to a gradual increase of Δ*G_m_*, according to Equation (1), generating the driving force for silica spinodal decomposition.

Coming to the CA-solvent–nonsolvent ternary phase diagram, in the organic/inorganic hybrid system, the kick-off positions A′ and A (in the polymer-solvent line, Figure 1B) are closer to the solvent vertex (if TEOS, water and acid are considered solvents). During the preparation of the casting solution, the formamide-silica spinodal decomposition occurs (which is not represented in the polymer–solvent–nonsolvent ternary phase diagram). After casting, acetone (the most volatile solvent) partially evaporates. Due to their solubility in acetone, the skinny membrane top layer risks being enriched in silica and unreacted TEOS (relatively to the global membrane composition). In the next step, formamide (solvent)–water (nonsolvent) exchange. TEOS (unreacted or hydrolyzed) will be forsaken into the water bath (due to TEOS-water immiscibility). So unreacted or hydrolyzed TEOS molecules stay in the hybrid membrane, allowing for sol–gel hydrolysis and condensation reactions to proceed. Those retarded sol–gel reactions, if accomplished, promote compositional heterogeneity in the membrane. TEOS-formamide immiscibility may reduce the hetero-condensation yield (by hindering the contact between TEOS and CA polymer molecules) but, at the same time, promote a homogeneous silica distribution in the hybrid membrane (through the silica network rupture by spinodal decomposition) inhibiting (or at least strongly reducing) the formation of silica clusters. 

In the present work, a novel protocol for the fabrication of accurate and reproducible monophasic hybrid CA/SiO_2_ ultrafiltration (UF) membranes with improved structural homogeneity (by preventing the formation of silica clusters) is described in detail. Two different membrane series were produced (series one and series two): each series exhibited a constant formamide/acetone ratio and increasing silica content. The membranes in series one were prepared with a lower formamide/acetone ratio. The morphology, chemical structure, mechanical properties and permeation performance of the membranes in the two series are evaluated by SEM, FTIR-ATR, mechanical tests (tensile tests) and the permeation of pure water. 

## 2. Experimental Procedure

### 2.1. Materials

Membranes were synthesized with cellulose acetate (CA, C_6_H_7_O_2_(OH)_3_, ~30,000 g·mol^−1^, reagent grade ≥ 97%, esterification degree ~40%), tetraethoxysilane (TEOS, Si(OC_2_H_5_)_4_, 208.33 g·mol^−1^, reagent grade ≥ 98%), and formamide (CH_3_NO, 45.02 g·mol^−1^, ≥99.5%) purchased from Sigma-Aldrich (Steinheim, Germany) and acetone (C_3_H_6_O, 58.08 g·mol^−1^, ≥99.6%) and nitric acid (HNO_3_, 63.01 g·mol^−1^, 1.39 g·mL^−1^ at 20 °C, 65% *v*/*v*) purchased from ABSOLVE (Jose Manuel Gomes Dos Santos LDA).

Membranes were dried with Propan-2-ol (25% *v*/*v*, 50% *v*/*v*, 75% *v*/*v*, 100% *v*/*v*) (anhydrous, 99.8%) and n–hexane (25% *v*/*v*, 50% *v*/*v*, 75% *v*/*v*, 100% *v*/*v*) (95%) purchased from Fisher Scientific, (Geel, Belgium). Bi-distilled water (H_2_Od) (conductivity 0–2 µS·cm^−1^, pH 5.8–6.5) and pure deionized (DI) water were obtained at the Laboratory of Membrane Processes, IST, Lisbon, Portugal.

All chemicals used in the synthesis and drying of the membranes were used without further purification.

### 2.2. Membranes Synthesis 

Eight different membrane compositions were studied, enclosed in two series—series one and series two. In each series, the silica amount increased from 0 wt.% up to 30 wt.% (with increments of 10 wt.%), while the solvent composition was kept constant within each series (formamide/acetone ratio equals 0.57 wt.% in series one and 0.73 wt.% in series two). Series one kept the formamide/acetone ratio reported in the authors’ previous work [7,8,10,11], while series two trigger a new formamide/acetone ratio.

Membrane compositions are shown in Table 1 (weight basis). Membranes’ acronyms indicate the membranes’ mass composition (casting solution CA/silica weight ratio).

Based on some of the authors’ previous work [7,8,10,11], a novel experimental protocol was developed and optimized to fabricate accurate, reproducible integrally skinned asymmetric monophasic hybrid CA/SiO_2_ UF membranes. 

**Figure 3:**
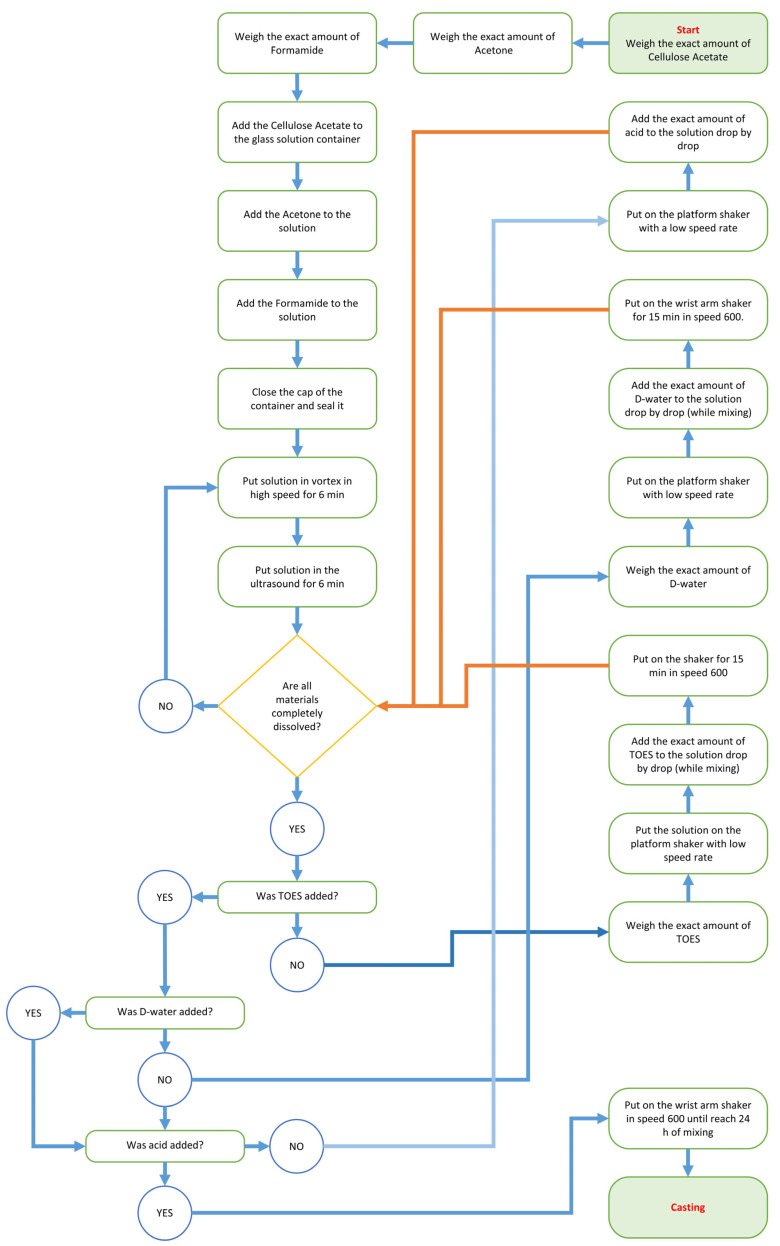
Flowchart of hybrid membranes preparation.

The membranes’ casting solutions were prepared in two stages. The first one consists in mixing the polymeric system (CA, formamide, and acetone) until a transparent and homogeneous solution is obtained. In the second stage (starting only after 2.5–4 h of mechanical mixing), the silica sol–gel system was introduced in the polymeric casting solution. TEOS was the first compound added, deionized water the second one, and nitric acid the third, to boost hydrolysis during the casting solution homogenization step. All the sol–gel compounds were added drop by drop. A total 24 h mixing/homogenization period was adopted under vigorous mechanical stirring (mechanical arm, magnetic field, vortex) and ultrasound bath (methods used alternately and sequentially, see flowchart in Figure 3).

The hybrid casting solutions were then cast onto a glass plate at room temperature, and a liquid film was produced by sliding a 250 μm casting knife. After a waiting time of 30 s (to allow acetone evaporation), the glass plates (subtract plus polymeric film) were quenched into a gelation bath (ice-cold deionized water, the nonsolvent phase). 

After a residence time of approximately 24 h in the gelation bath, the membranes were detached from the glass plate, washed thoroughly with deionized water to remove any solvent traces, and finally stored in deionized water at 4 ± 1 °C. 

Each casting solution grants eight membrane sheets (20 cm (width) × 30 cm (height) × 250 µm (thickness)). 

### 2.3. Membranes’ Drying 

The membranes’ drying step is mandatory when membranes’ preservation (pore size and pore distribution) during storing, transport, and characterization is needed. Membranes were immersed in an aqueous glycerol solution (20% *v*/*v*) for 15 min according to the reported description by [4] (details in Annex I). After removal, the membranes were dried in the laboratory atmosphere. 

### 2.4. Membranes Characterization

Membranes were structurally (SEM and FTIR-ATR) and mechanically (tensile tests) characterized, and ultra-filtration performance (hydraulic permeability) was evaluated in a systematic way to confirm the accuracy and reproducibility of the synthesized membranes. All the tests, with the exception of the SEM analysis, were performed on wet membranes. 

For each membrane composition, five samples (from different membrane sheets produced in the same batch) were characterized in terms of surface and cross-section morphology, tensile tests, and pure water permeation performance. 

#### 2.4.1. Morphological Characterization

##### SEM

Membrane surface morphology and cross-section structure were analyzed by Field Emission Gun–Scanning Electron Microscopy (FEG-SEM) (JEOL 7001F JOEAL, Tokyo, Japan). SEM images of the top active layer surfaces and the bottom porous surfaces were taken at 5000× magnification, and the cross sections were taken between 2500× magnification.

Prior to being imaged, the membranes were cut (1 cm × 1 cm) and dried. Dried membranes were fractured in liquid nitrogen, mounted on a stub and sputter-coated with gold. Membranes’ (total) thickness and skin layer thickness were measured from FEG-SEM cross-section images with the ImageJ1.53t software. Five randomly selected zones from the entire cross-section images were measured for each membrane, and the mean thickness and standard deviation were calculated.

##### FITR-ATR

The two-membrane series is composed of organic CA-based and inorganic SiO_2_-based counterparts, differing in the silica content (within each series) and solvent ratios (between the two series). In each series, the effect of SiO_2_ on the hybrid membrane network is studied by comparing the spectra of membranes with increasing SiO_2_ content. The formamide/acetone ratio effect is evaluated by matching the spectra of membranes from the two series with the same SiO_2_ content.

FTIR-ATR spectra of the membranes from series one and series two were obtained and analyzed in the region 3750–700 cm^−1^. First, the FTIR-ATR wide spectra were analyzed to identify the types of bonds present. To study the incorporation of the silica component into the organic matrix, the FTIR region specific to Si bonds–namely ν_δ_(Si-O-Si) and ν_δ_(Si-O-C), that occurs at 950–1190 cm^−1^ were analyzed in greater detail.

Infrared spectra of randomly selected samples of each membrane composition were obtained with a Nicolet Magna IR System 5700 spectrometer (Nicolet Instrument Corp., Madison, WI, USA), using a Golden Gate MKII ATR accessory with a Ge crystal (Graseby Specac, Smyrna; sampling depth: 0.2–1.1 μm at 3750–700 cm^−1^). Each spectrum was obtained by averaging 64 scans with a resolution of 2 cm^−1^. The ν_δ_(Si-O-Si) bands for the different membranes, located between 950 and 1190 cm^−1^, were decomposed by curve-fitting with Gaussian bands (Levenberg–Marquardt algorithm, allowing variation in width, height, and position of the bands) after a baseline correction (subtraction of a straight line between two extreme wavenumbers of the region) and the number and starting position of the bands used in the fitting were obtained from the smoothed (Savitzky–Golay algorithm) second-derivative spectrum of the region. For each of the monophasic hybrid membranes, four individual peaks were found, and a non-linear least-squares fitting procedure was performed to obtain 100% Gaussian-shaped peaks. The quality of the fit was estimated through the evaluation of *χ*^2^ remained constant.

#### 2.4.2. Ultra-Filtration Performance

Permeation experiments were performed to characterize all membranes in terms of pure water hydraulic permeability (*Lp*). The laboratory setup used in the permeation experiments has been previously described [26](details in Annex II). Briefly, it consists of a flat cell unit with two detachable parts separated by a porous plate (membrane support) with a membrane surface area of 13.2 × 10^−4^ m^2^ (comprising five flat-cell units) that was used in the permeation experiments. Each flat-cell unit connects the water reservoir, with the possibility to adjust water pressure from zero up to 5 bar. In all the experiments, the feed temperature was kept constant at 25 °C. 

The hydraulic permeability of the membrane is obtained by the slope of the straight line of pure water permeate fluxes (*J_pw_*) as a function of the transmembrane pressure (∆*P*) defined as $J_{pw}/\Delta P$. The range of the transmembrane pressure used was from 1 bar to 5 bar.

Pure water mass flux (for each time interval) was calculated by using Equation (5) [27,28]:

(5) $$J_{pw}=\frac{Q}{\Delta t\cdot A}$$

where $J_{pw}$ is the pure water flux (kg.m^−2^·h^−1^), *Q* is the amount of permeates collected (kg), ∆*t* is the sampling time (h), and *A* is the membrane area (m^2^).

Before hydraulic permeation assays, all membranes were compacted for 5 h by circulating deionized water at a flow rate of 100 L·h^−1^ and at a transmembrane pressure of 5 bar. Compaction details are described in Annex II. The procedure to measure the $J_{pw}$ value was repeated three times for each water pressure condition and each specimen.

#### 2.4.3. Mechanical Performance

Membranes’ tensile properties (Young’s modulus and the yield point stress and strain, for a fixed elongation rate) were measured with Universal Testing Machine Instron^®^ 5544 (Instron^®^, Norwood, MA, USA) equipped with a 100 N Load Cell (Instron^®^, Norwood, MA, USA). For the synchronous acquisition of the strain, a Standard Video Extensometer (SVE 1, Instron^®^, Norwood, MA, USA) was employed, being previously calibrated using an Instron^®^ calibration frame. To simulate in-service conditions, the membranes’ tensile tests were performed in an aqueous bath (deionized water) using a BioPlus Temperature Controlled Bath system and the BioPuls Submersible Pneumatic Side Action Grips (Instron^®^, Norwood, MA, USA) at room temperature. 

The tensile tests were conducted with a constant elongation rate of 6 mm/min (crosshead displacement) based on some of the authors’ previous work [8]. The specimens were carefully placed in the pneumatic grips, and a pre-tension of approximately 0.15 MPa (~0.2 N) was applied to ensure a uniform stretch over the entire section area. Then the pre-tension was removed, and the tensile test was initiated. The membranes were elongated inside the deionized water bath until the rupture point (fracture) was reached.

The specimen’s preparation details and the procedure used to measure the membranes thickness are detailed in Annex III. If a failure occurred during the preparation or placement of the membranes in the grips, the specimen was removed, and the results discarded. A retest trial, with the same test parameters but using only the membranes with 70% SiO_2_ in both series (CA1-SiO_2_ 70/30 and CA2-SiO_2_ 70/30) produced in a different batch, was conducted two weeks later to evaluate the consistency of their mechanical properties. 

The experimental data were recorded using the software BlueHill 3 (Instron^®^, Norwood, MA, USA) with an acquisition frequency of 10 Hz. Mechanical properties were computed using in-house routines developed in MATLAB R2021a software (MathWorks^©^, Natick, MA, USA). Young’s modulus was computed as the ratio of the stress (engineering)-strain relationship for the elastic region using a linear interpolation with least-squares method.

#### 2.4.4. Statistical Analysis

Descriptive statistics were applied for all the evaluated parameters, namely mean, median and standard deviation. Kruskal–Wallis’s test (with a null hypothesis that each variable is similar between groups) was performed to compare the experimental results between different compositions of each series for the hydraulic permeability and mechanical tensile tests. A pairwise comparison considering a Bonferroni correction method for multiple tests was applied to infer the existence of statistical differences between compositions of each series. On its turn, the Mann–Whitney U test (with a null hypothesis that the distribution of each variable is the same across groups) was used to compare each variable between series directly (e.g., CA1/SiO_2_ 90/10 vs. CA2/SiO_2_ 90/10).

A confidence level of 95% was defined t’ evaluate the existence of statistically significant differences between groups. A *p*-value lower than 0.05 was used as the threshold to reject the null hypothesis, indicating that there are significant differences in the hydraulic permeability and mechanical properties (Young’s modulus, yield point stress and yield point strain) between the two groups being compared. All statistical tests were computed using IBM SPSS^®^ Statistics v28 (IBM Corp. ^©^, Armonk, NY, USA) and in-house routines developed in MATLAB (MathWorks^©^, Natick, MA, USA). The results of the statistical analysis, namely the *p*-value and *U*-value, are presented in Tables S1–S4 of Annexes II and III. The existence of significant differences between groups is also indicated at the top of the bar graphs with the (*) symbol, where (*), (**), and (***) represent a *p*-value lower than 0.01, 0.05, and 0.1, respectively.

## 3. Result and Discussion

A new methodology is proposed to produce sustainable skinny asymmetric monophasic CA/SiO_2_ UF membranes. Several experimental challenges have been over-striped in series one and series two membranes’ fabrication, namely the cast solution homogenization (due to the TEOS-formamide immiscibility, which brought additional challenges to experimental protocol).

### 3.1. Morphological Characterization

#### 3.1.1. SEM

**Figure 4:**
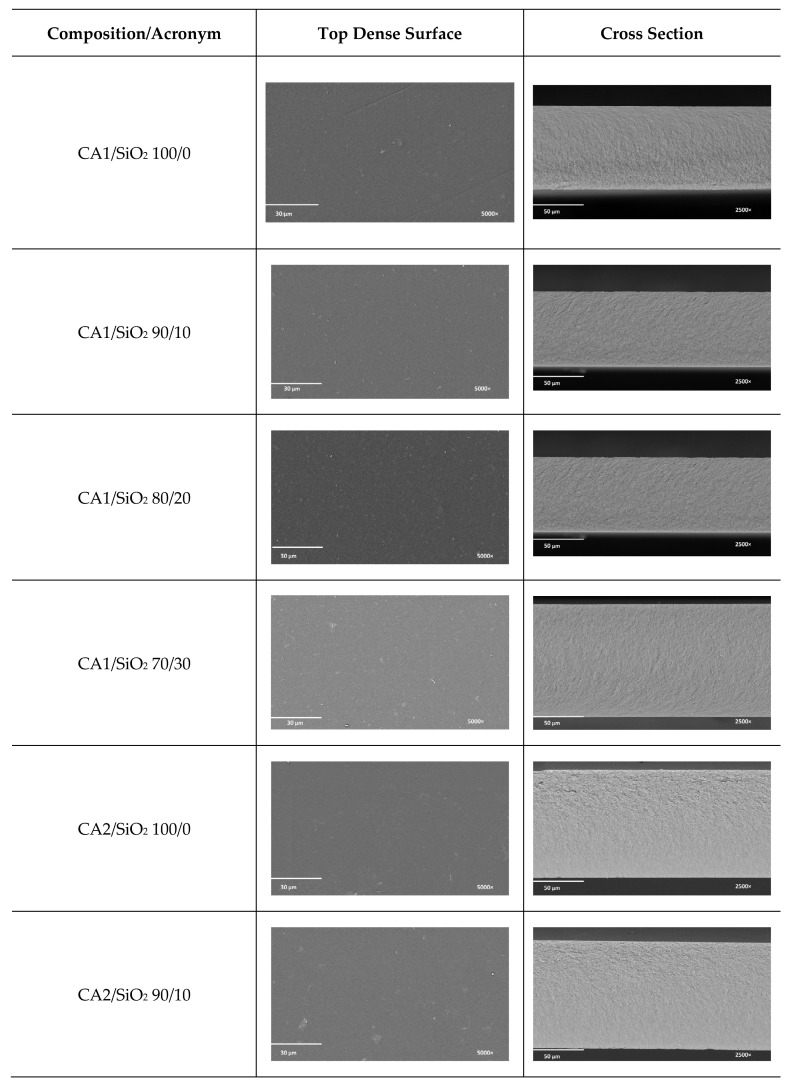

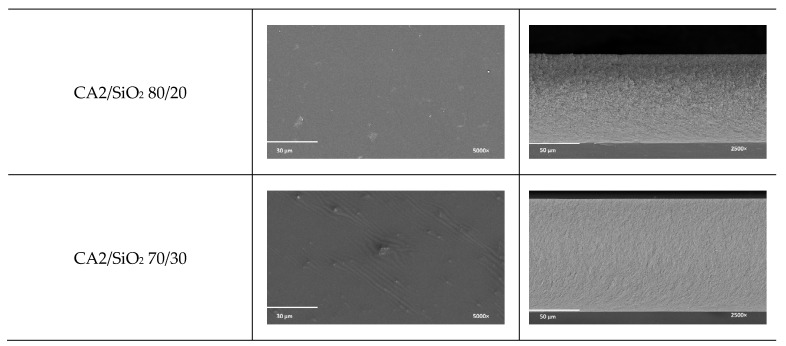
Top dense surface and cross-sectional images for the series one and series two membranes at 5000× and 2500× magnification.

SEM images of series one and series two (membrane’s top surfaces and cross-section morphologies) are shown in Figure 4. The SEM images of top surfaces clearly demonstrate smooth, non-porous, and dense membrane morphologies. The membrane’s cross-section SEM images clearly identify the membrane’s integral asymmetric nature, characterized by a thin skin denser layer on top (active layer) outlining a thicker and porous substructure (porous layer).

**Figure 5:**
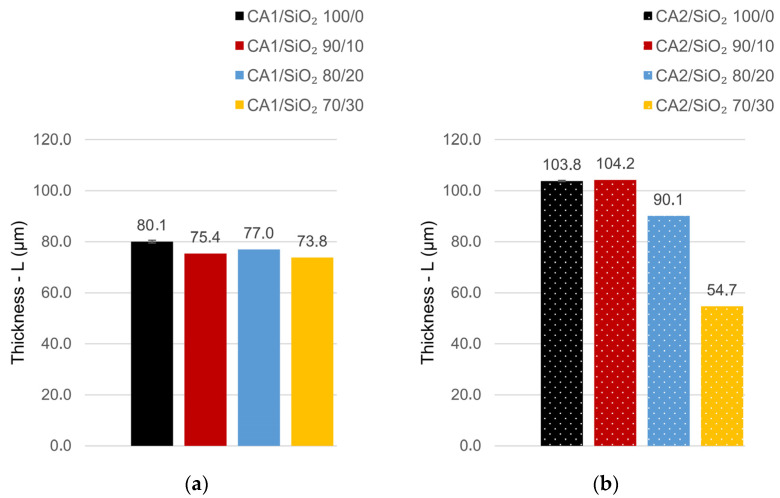
Total membrane thickness in (**a**) series one and (**b**) series two.

Figure 5 presents the SEM total membrane’s thickness as a function of series one and series two compositions. The total membrane thickness is studied in function of (i) the silica contents (discussed within each series, where the formamide/acetone ratio is kept constant) and (ii) the formamide/acetone ratio (series one versus series two) (Annex IV).

Let us start with the silica content. In series one, the silica content does not affect the membranes’ total thickness, which is not in accordance with some of the authors’ previous work for similar membrane compositions [11]. Though, in the present work, a new experimental protocol has been developed to improve CA/SiO_2_ membrane’s homogeneity, accuracy, and reproducibility when hydraulic permeability and mechanical performance are envisaged. In series one, membranes 90/10, 80/20, and 70/30 show an almost constant total thickness of 76.5 ± 3.5 µm, ranging between 74 and 77 µm (80 µm being the reference value, the thickness of pristine CA1/SiO_2_ 100/0). The total thickness of the membranes in series one seemed not to be affected by the amount of SiO_2_ present, which suggests a homogeneous and random accommodation of SiO_2_ tetrahedra in the organic CA network.

In series two (with a new formamide/acetone ratio), both pristine CA (CA2/SiO_2_ 100/0) and 90/10 (CA2/SiO_2_ 90/10) membranes exhibited very similar thickness values (108 µm and 104 µm, respectively). When silica content increases above 90/10 CA/SiO_2_ weight ratio, the overall membrane thickness decreases to half of the initial value, with the CA2/SiO_2_ 70/30 membrane exhibiting a total thickness of ~55 µm. The results for series two clearly show a new hybrid network tendency.

Now, let us look into the formamide/acetone ratio effect on total membrane thickness. Series one (with a formamide/acetone weight ratio of 0.57) exhibited lower total membrane thickness (between 77 and 74 µm), while series two (with a higher formamide/acetone weight ratio~0.73) showed higher total membrane thickness (between 104 and 90 µm, with an exception for membrane 70/30 with 55 µm). The pristine CA membrane exhibited 80 μm in series one versus 104 μm in series two. Our results are in alignment with [29], who observed an increase in the total thickness of CA membranes with formamide content (in similar polymeric casting solution compositions). Solvent exchange occurs between formamide (CA solvent) and water (CA nonsolvent), so the higher the formamide level, the larger the amount of solvent exchanged (Figure 1), promoting a higher porosity phase and, consequently, higher total membrane thickness, the reason why formamide is known by its swelling character. On another side, higher porosity implies higher surface area. This fact predicts distinct hydraulic permeability performance between membranes from series one and series two.

#### 3.1.2. FTIR-ATR

**Figure 6:**
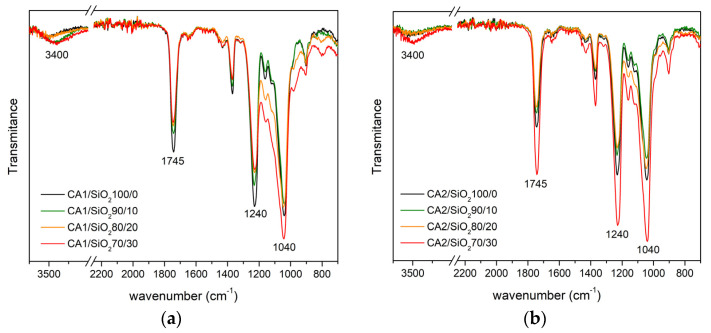
FTIR-ATR wide spectra (3750–700 cm^−1^) of the membranes in series one (**a**) and series two (**b**).

Figure 6a,b shows the FTIR-ATR wide spectra (3750–700 cm^−1^) obtained for the membranes of series one and series two, which are in qualitative agreement with other authors’ results for membranes with similar compositions [30,31] (Annex V). The most intense bands of the pure CA membranes (100/0, black lines) and the hybrid membranes (90/10, green lines, 80/20, orange lines, and 70/30, red lines) in both series one and series two are found at approximately 1040 cm^−1^, 1240 cm^−1^, and 1745 cm^−1^, and are attributed to ν_δ_(C-O-C), ν_δ_(C-O), and ν_δ_(C=O) vibrations, respectively. The broad band located at approximately 3400 cm^−1^ is attributed to the ν_s_(OH) of unacetylated OH groups (of the CA polymer), which has been studied by other authors in solvent dried CA membranes [30,31].

**Figure 7:**
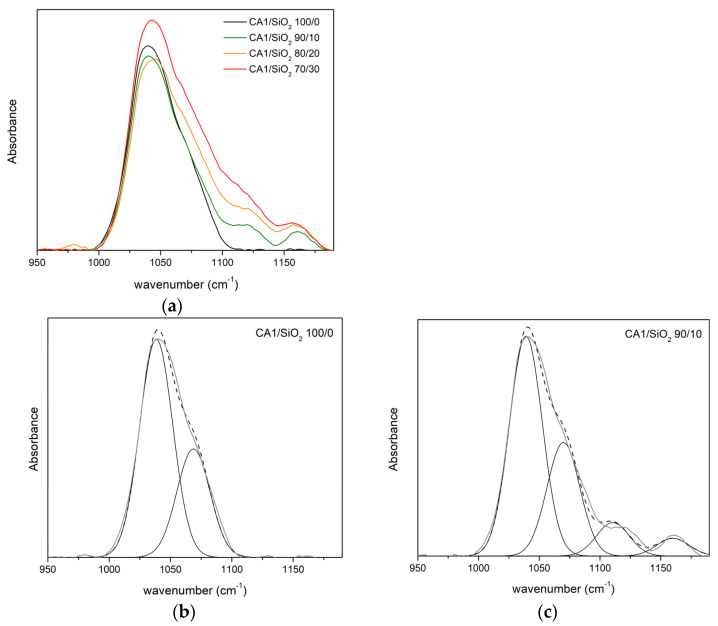

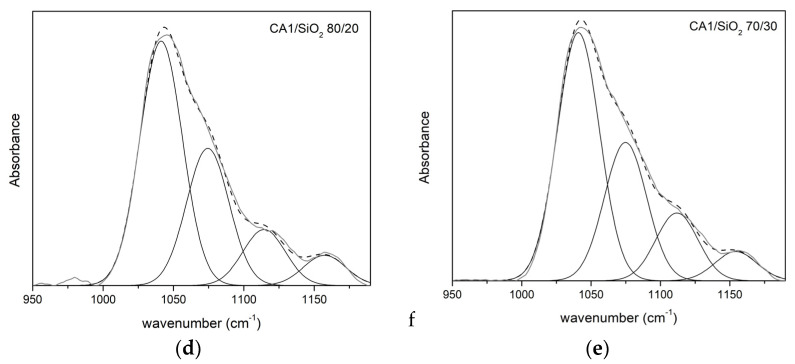
FTIR-ATR absorption spectra of the membranes from series one: CA1/SiO_2_ 100/0, 90/10, 80/20, and 70/30 membranes: (**a**) in the region 950–1190 cm^−1^ and (**b**–**e**) curve-fitting decomposition of the bands ν(C-O), ν_δ_(Si-O-Si), and ν_δ_(Si-O-C), present in the spectra obtained for: (**b**) CA1/SiO_2_ 100/0 membrane, (**c**) CA1/SiO_2_ 90/10 membrane, (**d**) CA1/SiO_2_ 80/20 membrane, and (**e**) CA1/SiO_2_ 70/30 membrane. Experimental results are shown by the thick grey line and the simulated band by the black dashed line.

**Figure 8:**
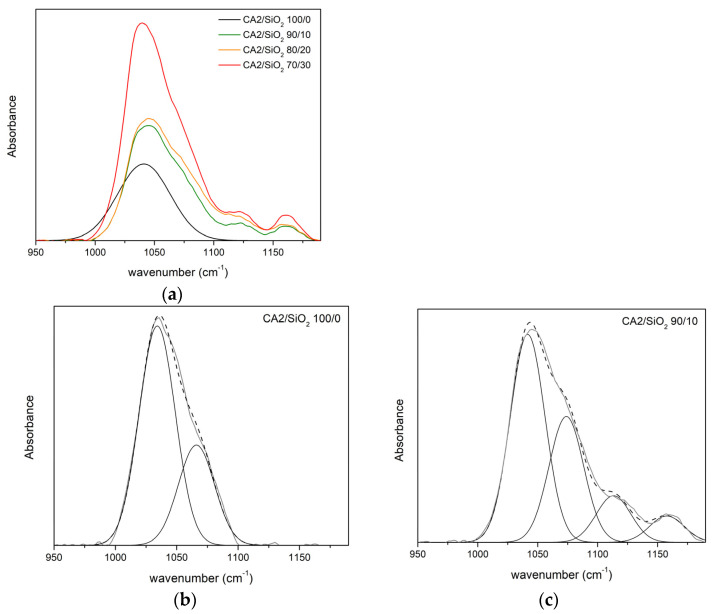

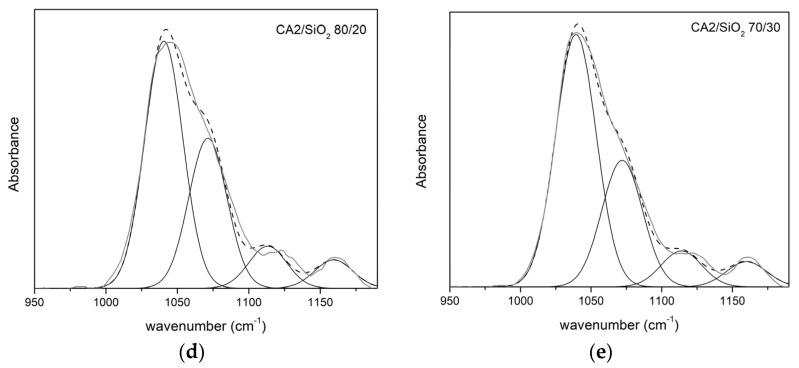
FTIR-ATR absorption spectra of series two membranes: CA2/SiO_2_ 100/0, CA2/SiO_2_ 90/10, 80/20, and 70/30: (**a**) In the region 950–1190 cm^−1^ and (**b**–**d**) curve-fitting decomposition of the ν_δ_(C-O), ν_δ_(Si-O-Si) and ν(Si-O-C) bands, present in the spectra obtained for: (**b**) CA2/SiO_2_ 100/0 membrane, (**c**) CA2/SiO_2_ 90/10 membrane, (**d**) CA2/SiO_2_ 80/20 membrane, and (**e**) CA2/SiO_2_ 70/30 membrane. Experimental results are shown by the thick grey line and the simulated band by the black dashed line.

Pristine CA membranes (in series one and series two) are taken as a reference for the FTIR-ATR studies. Figure 7 and Figure 8 show the FTIR-ATR absorption spectra of the hybrid membranes from series one and series two, in the region between 950 and 1190 cm^−1^. Figure 7a and Figure 8a show the spectra obtained after curve fitting of the pristine CA1 and CA2 membranes, respectively. In both spectra, two peaks located between 1034 and 1039 cm^−1^ and between 1066 and 1067 cm^−1^ are found and correspond to the ν_δ_(C-O) and ν_δ_(C-O-C) stretching vibrations of the CA1/SiO_2_ 100/0 and CA2/SiO_2_ 100/0 membranes [30,32,33,34].

In addition to the peaks corresponding to the ν_δ_(C-O) and ν_δ_(C-O-C) stretching vibrations, all the hybrid membranes from series one and series two also exhibit two peaks characteristic of SiO_2_, assigned to ν_δ_(Si-O-C) and ν_δ_(Si-O-Si), which were further analyzed by peak decomposition. The position, shape, and relative peak area of the vibrational bands of the monophasic hybrid membranes were analyzed and compared to the pristine CA membranes from each series (CA1/SiO_2_ 100/0 and CA2/SiO_2_ 100/0).

##### Series One

In series one, Figure 7b–e show the curve-fitting decomposition of the bands located between 950 and 1190 cm^−1^ correspondent to the vibration bands of ν_δ_(C-O) (first peak between 1031 and 1041 cm^−1^), ν_δ_(C-O-C) (second peak found between 1066 and 1075 cm^−1^), ν_δ_(Si-O-C) (third peak found between 1111 and 1114 cm^−1^), and ν_δ_(Si-O-Si) (forth peak found between 1154 and 1060 cm^−1^). Table 2 shows the frequency and relative area of each peak for the membranes of series one.

Figure 7c–e show the peak corresponding to the ν_δ_(C-O) vibration for the membranes CA1/SiO_2_ 90/10, 80/20, and 70/30. When compared to the reference membrane (CA1/SiO_2_ 100/0, Figure 7b), we can observe that the introduction of only 10 wt.% of SiO_2_ was observed to decrease the ν_δ_(C-O) peak area from 67% to 51%. Further increase of SiO_2_ content, up to 20 and 30 SiO_2_ wt.%, results in a band area decrease, from 67% to 52% and 57%, respectively.

Figure 7c–e also show a strong peak between 1070 and 1075 cm^−1^, which can be attributed to the ν_δ_(C-O-C) stretching band found in the pristine CA1 membrane or to the ν_δ_(Si-O-Si) stretching vibration [35,36,37,38,39]. Since the percentages attributed to this second peak (25–33%) are very close to the percentage of the pristine membrane (33%), it is envisioned that this contribution is due to the C-O-C groups of cellulose acetate rather than Si-O-Si groups. Both the intense peak and shoulder increase with the SiO_2_ content, indicating the formation Si-O-Si network.

All the hybrid membranes exhibited a third peak located between 1111 cm^−1^ and 1114 cm^−1^, attributed to the ν_δ_(Si-O-C) [37,38,39,40]. The area of this peak increases with the silica content, from 9% to 12% and 14% in CA1/SiO_2_ 90/10, 80/20, and 70/30 membranes, respectively. The increase of ν_δ_(Si-O-C) peak area with SiO_2_ content confirms the establishment of a covalent linking between the organic (CA) and inorganic (SiO_2_) counterparts. The hybrid membranes exhibit a fourth peak located between 1154 and 160 cm^−1^ corresponding to the ν_δ_(Si-O-Si), indicating the formation of the Si-O-Si network. The area of this peak decreases with the increase of silica content and, therefore, it is concluded that the increase of SiO_2_ from 10 to 30 wt.% decreases the formation of Si-O-Si networks.

Taking into account the third and fourth peaks found for the hybrid membranes, there is an indication that although some of the Si-OH groups from the silica precursor (homo-)condense with other Si-OH groups to form Si-O-Si network (eventually creating nano/micro SiO_2_ clusters), there are other silanol groups that (hetero-)condense with -C-O groups of the CA polymer, originating the hybrid Si-O-C bond. Moreover, the number of the Si-O-C groups increases with the SiO_2_ content, being highest for the CA1/SiO_2_ 70/30 membrane.

##### Series Two

Figure 8a shows the 950–1200 cm^−1^ FTIR-ATR spectra of series two membranes. Figure 8b shows the spectra obtained after curve fitting of the pristine CA membrane (CA2/SiO_2_ 100/0). As observed for the pure C membrane in series one, two peaks located at 1034 and 1066 cm^−1^ (68 and 32% area, respectively) are found and attributed to the ν_δ_(C-O) and ν_δ_(C-O), respectively [30,32,33,34].

Figure 8c–e show the curve-fitting decomposition of the band located between 950 and 1190 cm^−1^ correspondent to ν_δ_(C-O) (first peak between 1034 and 1042 cm^−1^), ν_δ_(C-O-C) (second peak found between 1066 and 1074 cm^−1^), ν_δ_(Si-O-C) (third peak found between 1123 and 1124 cm^−1^), and ν_δ_(Si-O-Si) (forth peak found at 1160 cm^−1^). Table 3 shows the frequency and relative area of each peak for the series two membranes.

In Figure 8c–e, the peak appearing at 1041 cm^−1^ decreases when compared to the one in the reference membrane (CA2/SiO_2_ 100/0). The introduction of only 10, 20, and 30 wt.% of SiO_2_ in the pure CA membrane decreases the area of ν_δ_(C-O) from 68% to 46%, 51%, and 57%, respectively.

The second peaks found in the spectra of the hybrid membranes (Figure 8c–e) between 1071 and 1072 cm^−1^ are attributed to the ν_δ_(C-O-C). As was said for the membranes of series one, peaks appearing in this region can also be attributed to ν_δ_(Si-O-Si), but because the areas of the peaks are similar to the one found in the pristine CA membrane of series two (32%) and because the CA content is always much higher than the SiO_2_ content in all membranes, it is estimated that this peak appears in all membranes due to the C-O-C groups of CA. The third peaks found in Figure 8c–e, located at 1123 cm^−1^ and 1124 cm^−1^, are attributed to the ν_δ_(Si-O-C) [37,40]. The fourth and last peak appearing at 1160 cm^−1^ for the CA2/SiO_2_ 90/10, 80/20, and 70/30 membranes is attributed to ν_δ_(Si-O-Si) [35,37,38,39,40]. The fourth peak characteristic of the ν_δ_(Si-O-Si) does not seem to be influenced by the amount of SiO_2_ (appearing in approximately the same position and with the same intensity—6%) [37,40].

The area of the peak attributed to ν_δ_(Si-O-C) decreases from 16% for the CA SiO_2_ 90/10 membrane to 11% and 8% for the CA2/SiO_2_ 80/20 and CA2/SiO_2_ 70/30 membranes, respectively. This gives evidence that, for series two, in contrast to what was observed in series one, the number of Si-O-C bonds decreases with the SiO_2_ content, being accompanied by an increase in the number of free C-O groups (predominantly present in the pristine CA membrane). As was seen for the membranes in series one, there is an indication that some Si-OH groups of the SiO_2_ precursor (homo-)condense with other Si-OH groups to form the Si-O-Si network, while other silanol groups (hetero-)condense with the C-O group of the CA polymer to form hybrid network Si-O-C.

To summarize, at the molecular level, a hybrid monophasic network is more prone to occur in series one (with an increasing value of Si-O-C with SiO_2_ content) than in series two (where Si-O-C decreases with SiO_2_ content).

### 3.2. Ultrafiltration Performance

**Figure 9:**
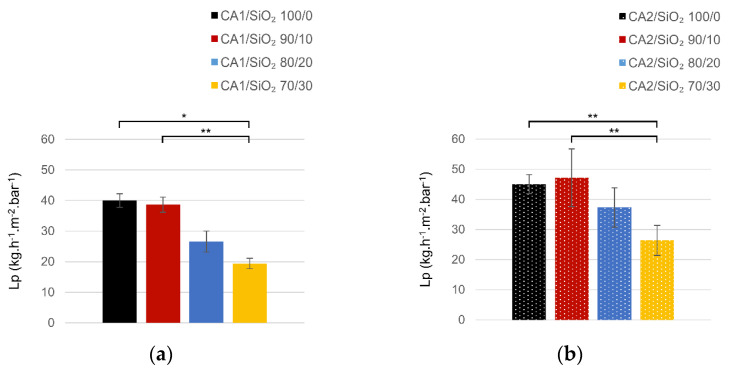
Hydraulic permeability *Lp* values (kg·h^−1^·m^−2^ bar^−1^) in (**a**) series one and (**b**) series two (* *p* < 0.01; ** *p* < 0.05).

The hydraulic permeability *Lp* values for the two membranes series are plotted in Figure 9. We will start by comparing the solvent system. In series one the maximum value for *Lp* was found for the pristine CA membrane (40 kg·h^−1^·m^−2^ bar^−1^). The presence of SiO_2_ promotes a monotonous decrease in *Lp*, which reduces from 40 kg·h^−1^·m^−2^ bar^−1^ (pristine CA membrane) until 19 kg·h^−1^·m^−2^ bar^−1^ (for 30 wt.% of SiO_2_).

Series two reveals a slightly different behavior as predicted from the SEM (higher) membrane total thickness. First, the *Lp* value for the pristine CA membrane is higher than for the pure CA membrane in series one (45 kg·h^−1^·m^−2^ bar^−1^ for series two, 40 kg·h^−1^·m^−2^ bar^−1^ for series one). Secondly, *Lp* exhibited a maximum of 47 kg·h^−1^·m^−2^ bar^−1^ for a 10 wt.% of SiO_2_ content. And finally, for silica content higher than 10 wt.%, a monotonous decrease in *Lp* was observed, with the lowest value, 25 kg·h^−1^·m^−2^ bar^−1^, for the membrane containing 30 wt.% of SiO_2_.

When comparing *Lp* values, membranes in series two exhibit higher *Lp* values than the ones in series one. This is due to the solvent system used in the casting solutions of each of the series, which evidences that a higher formamide/acetone ratio results in membranes with higher affinity towards the water and hence higher *Lp* values. This fact is in accordance with the SEM morphological characterization predictions, as higher total membrane thickness is associated with higher porosity and higher surface area. The higher number of -Si-OH groups present in series two membranes (evidenced in FTIR-ATR analysis, lower hetero-condensation in series two) are prone to higher water affinity.

Looking into the silica’s role, when comparing *Lp* to the overall thickness of the membranes in series one, it is obvious that they do not correlate as thickness remained essentially the same (between 74 and 77 µm), while *Lp* seems to decrease with the increase of SiO_2_ content. The silica presence does not enhance the hydraulic permeability of the membranes, probably due to the predominant hetero-condensation present in series one membranes’ group and consequent quite homogeneous silica tetrahedra distribution all along the CA network.

The membranes in series two containing 0 wt.% and 10 wt.% of SiO_2_ had essentially the same thickness (~104 µm). For the membrane containing 20 wt.% of SiO_2_, the total thickness decreased to 90 µm, and for the membrane containing 30 wt.% of SiO_2_, the thickness further decreased to 55 µm. The pure CA membrane and the CA2-SiO_2_ 90/10 membrane have very similar values of *Lp* (45 and 47 kg·h^−1^·m^−2^ bar^−1^). When SiO_2_ content was 20 wt.% and 30 wt.% the *Lp* values decreased to 37 and 26 47 kg·h^−1^·m^−2^ bar^−1^, respectively. Silica contents higher than 10 wt.% lead to membranes with smaller (total) thicknesses along with lower values of *Lp*. In series two the porosity and surface area play key roles when *Lp* is concerned. In series two the role of surface silanol groups looks smoothed in comparison to series one.

Considering what was found for the chemical structure of the membranes in series two, where the amount of bonded carbonyl groups increased with silica content, we can conclude that membranes where hydrogen bonding is higher result in smaller membrane thicknesses and lower values of *Lp*.

### 3.3. Mechanical Performance

The mechanical performance of the hybrid membranes was studied, addressing the membranes’ silica content and formamide/acetone volume ratio. The experimental stress–strain curves for series one and series two membranes (specimens cut from different regions of the membrane sheet and between different sheets produced from the same or distinct batches) are presented in Annex III.

Stress–strain relationships obtained for the elastic region were consistent between trials, implying that both Young’s modulus and yield strength are consistent for membranes produced in the same batch. In contrast, the failure region exhibited great variability in the rupture point, which is attributed to micro-fractures induced during the preparation of the specimens. In fact, when samples with (visible) micro-fractures were discarded (retest trials), no significant differences in the failure point were observed. Moreover, from the analysis of the stress–strain curves, it is possible to infer that the ultimate tensile strength occurs near the failure point. It is also possible to observe that the membranes, in general, present a ductile behavior, sustaining a large deformation in the plastic regimen (retest trials: CA1-SiO_2_ 70/30–10.1% (1.47 MPa), CA2-SiO_2_ 70/30–12.8% (1.43%)).

**Figure 10:**
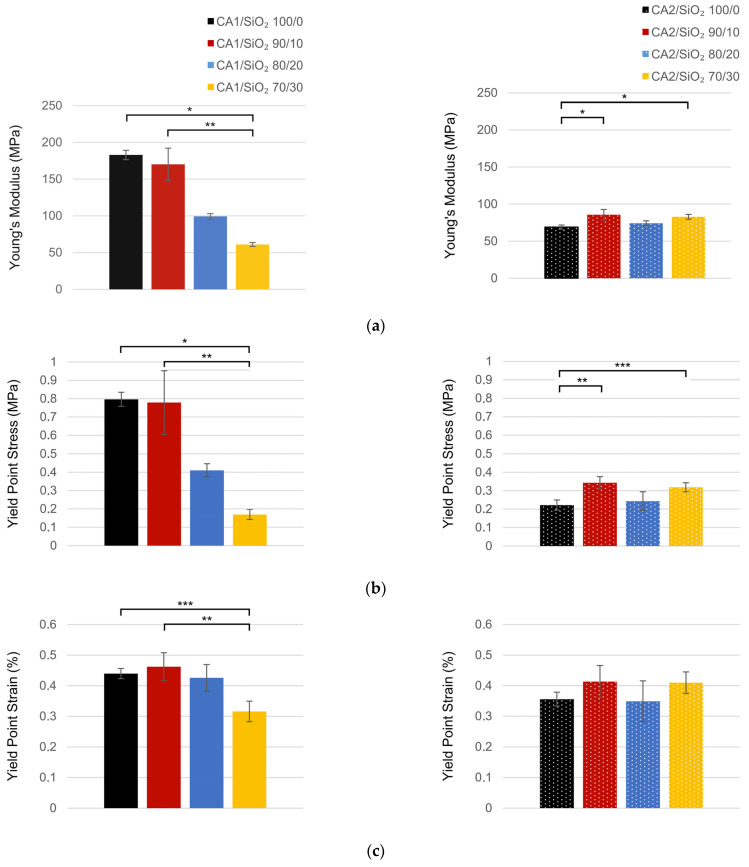
Mechanical properties for series one (**left**) and series two (**right**): (**a**) Young’s modulus (**top**); (**b**) stress at yield point (**middle**); and (**c**) strain at yield point (**bottom**) (* *p* < 0.01; ** *p* < 0.05; *** *p* < 0.1).

Regarding the silica content, each series was studied independently. For series one, Young’s modulus decreased with the SiO_2_ amount (Figure 10a). A decrease of approximately 6.9% was observed between pristine CA and CA1/SiO_2_ 90/20, followed by a decrease of 41.7% for CA1/SiO_2_ 80/20 and, finally, a decrease of 38.5% between the CA1/SiO_2_ 80/20 and CA1/SiO_2_ 70/30. Similarly, in series one, the yield stress was observed to increase with SiO_2_ content (see Figure 10c), denoting that higher axial loads and stress values are needed to stretch the membranes and to enter in plastic deformation for the membranes with higher SiO_2_ ratios. Despite this behavior, the strain at which each type of membrane entered in plastic regimen was similar between the different formulations, in particular for the ones with 100/0 (0.44%), 90/10 (0.46%), and 80/20 (0.43%) (see Figure 10e). A large reduction was only observed in the membrane with 70% of SiO_2_ composition (0.32%), indicating that this type of membrane sustains lower deformation ratios in the elastic domain. The inorganic bonds are less elastic than the organic counterparts. In series one, a true hybrid network with a homogeneous distribution of the silica tetrahedra all along the CA network is observed, which explains the decrease in Young’s modulus with silica.

In contrast, series two presents an almost constant Young’s modulus (see Figure 10b), indicating that the presence of silica does not affect the mechanical performance of the membranes. An increase of 24% in Young’s modulus was observed between pristine CA and CA2/SiO_2_ 90/10, followed by a decrease of 13.5% in the CA2/SiO_2_ 80/20 composition and an increase of 11.8% between the CA2/SiO_2_ 80/20 and 70/30. A similar trend was observed for the yield strength, presenting values ranging from 0.22 MPa for the pristine CA composition to 0.34 MPa for the CA2/SiO_2_ 90/10 composition (see Figure 8d). Regarding the yield strain, similar values were seen between different compositions (Figure 10f), denoting that the strain at which the membranes entered in the plastic domain is also not affected by the differences in SiO_2_ level for the tested formulations. Here almost two phases system is formed, with micro-silica clusters having a negligible effect on the tensile properties.

The comparison of the mechanical parameters between the two series enables us to conclude that the solvents ratios used during their preparation have a strong influence on the membranes’ mechanical properties. In particular, series one membranes with lower SiO_2_ values presented a stiffer behavior when compared with series two. A reduction of 62% of Young’s modulus (*p* < 0.01) and 72% of the stress (*p* < 0.01) needed to enter in the plastic domain was observed between the pristine CA membranes of series two in relation to series one. A reduction of 49.7% and 56.0% were also observed, respectively, for Young’s modulus (*p* = 0.029) and yield point stress (*p* = 0.029) in the membranes with a 90/10 SiO_2_ ratio. Moreover, it is important to highlight that Young’s modulus obtained for all formulations of series two was within the range of values observed for the 70/30 and 80/20 compositions of series one. Despite these differences, the values obtained for the strain at the yield point were equivalent between series, denoting that although the elasticity is different, the strain needed to enter in plastic regimen is similar. The major difference was observed in the 70/30 SiO_2_ ratio, in which an increase of 29.7% in the yield point stress value was observed in the series two membranes (*p* < 0.01).

These differences in the mechanical properties between series indicate that the membranes’ chemical structure could be different. The higher homogeneity in series two suggests that silica did not affect the CA network. This fact is in accordance with the existence of a two phases system (nano/micro-composite), where the silica phase (silica micro-clusters) is minimal. On the contrary, in series one, silica being homogeneously bounded to the CA main organic network originates a new monophasic system with particular properties.

When compared with the results presented in [8], higher Young’s modulus values were obtained for the series one formulations with lower SiO_2_ ratios. On its turn, the values obtained for series two were similar to the ones obtained in this work. However, no direct comparisons can be made, since in Andrade et al. study the membranes were not tested in an aqueous solution, which could have influenced the mechanical properties of the specimens.

Despite the adoption of a “dog bone” shape for the tensile specimens, several samples broke earlier than expected (lower strain ratio at failure point) or failed at the grips or shoulder region. As the first hypothesis, the non-homogeneity of the membrane, resulting in different mechanical properties on the central or lateral parts of it, was considered. This hypothesis was posteriorly refuted on the retest trials since no differences were observed for Young’s modulus, yield strength, ultimate tensile strength, and failure point between specimens cut from different regions of the sheet. This point is particularly relevant as it indicates that in terms of tensile properties, the membranes appear to be homogenous along the sheet.

The second hypothesis is based on the eventual introduction of micro-fractures during the preparation of the specimens. This was analyzed during the retrial tests and partially corroborated by the results. Cutting the membranes proved to be a more difficult procedure than expected, as any abnormal movement of the scalpel or the 3D cutting cast induced some failures in the specimen. Although these small defects did not affect the results for the elastic region, they explained the variability seen on the failure region for the first trials, namely the values obtained for the ultimate tensile strength and failure point. Moreover, even considering only the specimens that did not present any visible problems, some failed at the grips due to the deformations induced by the clamps, indicating that some changes should be considered in the future in the dimensions of the specimens, namely the reduction of the gauge width, or in the cutting process to reduce the ratio of rejected samples.

Nevertheless, it is important to note that the variability reported within the failure region or between batches is not problematic. Considering the final application of these membranes, they are not expected to work on the plastic regimen as the velocity of the fluid is considerably low.

## 4. Conclusions

A novel protocol to produce sustainable integral asymmetric CA/SiO_2_ membranes was optimized, and a highly homogeneous hybrid system (without the presence of micro-domains/clusters) was produced. Two formamide/acetone ratios were also studied.

In the series with the lowest formamide/acetone ratio (series one), a true monophasic system was obtained where silica tetrahedra are proved (by FTIR-ATR) to be randomly bounded to the CA network (higher amount of -Si-O-C- bonds, scarily less -Si-O-Si-). The hybrid network membrane (at the molecular level) exhibited lower hydraulic permeability due to the lower surface silanol groups. Higher Young’s modulus values were obtained as a new organic/inorganic network was formed. In the series with the highest formamide/acetone ratio (series two), a micro/nanocomposite structure was formed. Here, surface silanol groups correspond to high hydraulic permeability rates, and the biphasic matrix contributes to a lower Young modulus.

SEM images depict the asymmetric structure of the membranes in both series, where the active layer is characterized by a thickness of less than 1 µm. A higher formamide/acetone ratio (series two) leads to membranes with larger total thicknesses and a more porous sub-structure.

The experimental protocol presented in this work (in addition to a severe solvent ratios) plays a determinant role in the final properties of the monophasic hybrid CA/SiO_2_ membranes. These membranes exhibit interesting features that become potentially useful for chemical, pharmaceutical, and biomedical applications.

## Figures and Tables

**Table 1:** Casting solution formulation for series one (CA1/SiO_2_) and series two (CA2/SiO_2_) (weight basis).

Series One	Composition (g)	CA1/SiO_2_ 100/0	CA1/SiO_2_ 90/10	CA1/SiO_2_ 80/20	CA1/SiO_2_ 70/30
Polymer	CA	17.00	15.80	14.50	13.10
Solvent	Formamide	30.00	27.90	25.60	23.10
Solvent	Acetone	53.00	49.20	45.20	40.90
Silica precursor	TEOS	-	6.10	12.60	19.50
Reactant	H_2_O	-	1.00	2.10	3.40
Catalyst	HNO_3_	-	pH~2	pH~2	pH~2
TEOS/CA	TEOS/CA	-	0.39	0.87	1.49
CA/(solvent)	CA/(formamide + acetone)	0.20	0.20	0.20	0.20
Formamide/Acetone		0.57	0.57	0.57	0.57
**Series Two**	**Composition (g)**	**CA2/SiO_2_ 100/0**	**CA2/SiO_2_ 90/10**	**CA2/SiO_2_ 80/20**	**CA2/SiO_2_ 70/30**
Polymer	CA	17.00	17.00	17.00	17.00
Solvent	Formamide	35.00	35.00	35.00	35.00
Solvent	Acetone	48.00	48.00	48.00	48.00
Silica precursor	TEOS	-	6.55	14.70	25.30
Reactant	H_2_O	-	1.00	2.10	3.40
Catalyst	HNO_3_	-	pH~2	pH~2	pH~2
TEOS/CA	TEOS/CA	-	0.39	0.86	1.49
CA/(solvent)	CA/(formamide + acetone)	0.20	0.20	0.20	0.20
Formamide/acetone		0.73	0.73	0.73	0.73

Note: each TEOS unit originates one silica unit (1:1 basis).

**Table 2:** Series one: frequency (ν) and relative area (A %) of the bands ν_δ_(C-O), ν_δ_(Si-O-Si), and ν_δ_(Si-O-C).

	ν_δ_(C-O)		ν_δ_(C-O-C)		ν_δ_(Si-O-C)		ν_δ_(Si-O-Si)	
CA1/SiO_2_	ν (cm^−1^)	A (%)	ν (cm^−1^)	A (%)	ν (cm^−1^)	A (%)	ν (cm^−1^)	A (%)
**100/0**	1038	67	1069	33	-	-	-	-
**90/10**	1039	51	1070	33	1111	9	1160	7
**80/20**	1041	52	1074	29	1114	12	1157	6
**70/30**	1031	57	1075	25	1112	14	1154	4

**Table 3:** Series two: frequency (ν) and relative area (A %) of the bands ν_δ_(C-O), ν_δ_(Si-O-Si), and ν_δ_(Si-O-C).

	ν_δ_(C-O)		ν_δ_(C-O-C)		ν_δ_(Si-O-C)		ν_δ_(Si-O-Si)	
CA2/SiO_2_	ν (cm^−1^)	A (%)	ν (cm^−1^)	A (%)	ν (cm^−1^)	A (%)	ν (cm^−1^)	A (%)
**100/0**	1034	68	1066	32	-	-	-	-
**90/10**	1041	46	1071	32	1123	16	1160	6
**80/20**	1042	51	1074	31	1123	11	1160	6
**70/30**	1039	57	1072	29	1124	8	1160	6

## Data Availability

Not applicable.

## Supplementary Materials

The following supporting information can be downloaded at: https://www.mdpi.com/article/10.3390/membranes13030346/s1, Annex I. Membrane Drying Process; Annex II Ultrafiltration experimental set-up and compaction; Annex III Mechanical Properties; Annex IV. SEM image analysis; Annex V. IR assignments; Figure S1: Flowchart of Membrane’s Drying; Figure S2: FTIR-ATR of pristine CA membranes (wet and dry); Figure S3: Ultrafiltration experimental set up; Figure S4: Compaction curves for series1 and series2; Figure S5: (a) Experimental setup used for the acquisition of the mechanical properties of the series1 and series2 membranes (b) Representation of the specimen dimensions used during the tensile tests; Figure S6: Stress-strain curves for the series CA1 with a SiO_2_ composition of: (a) 100% (top left); (b) 90% (top right); (c) 80% (bottom left); (d) 700% (bottom right); Figure S7: Stress-strain curves for the series CA2 with a SiO_2_ composition of: (a) 100% (top left); (b) 90% (top right); (c) 80% (bottom left); (d) 700% (bottom right); Figure S8: Stress-strain curves for the series CA1 (top) and CA2 (bottom) with a SiO_2_ composition of 70% (retest trials). Left charts represent the stress-strain relationship for specimens cut from the lateral side of the membrane sheets and the right ones from the medial part; Table S1: Significance values (*p*-value) adjusted by the Bonferroni correction for multiple tests between the different SiO_2_ ratios of each series for the hydraulic permeability (Kruskal–Wallis test with pairwise comparisons for multiple tests); Table S2: Significance (*p*-value) and U-test values between series for a fixed SiO_2_ ratio for the hydraulic permeability (Mann–Whitney U Test); Table S3: Significance values (*p*-value) adjusted by the Bonferroni correction for multiple tests between the different SiO_2_ ratios of each series for the young’s modulus, yield stress and yield strain parameters (Kruskal–Wallis test with pairwise comparisons for multiple tests); Table S4: Significance (*p*-value) and U-test values between series for a fixed SiO_2_ ratio for the young’s modulus, yield stress and yield strain parameters (Mann–Whitney U Test); Table S5: Summary of images used to study the total thickness of series 1 membranes, and associated data; Table S6: Summary of images used to study the total thickness of series 2 membranes, and associated data; Table S7: Water IR assignments in CA membranes; Table S8: Chemical properties of -COOH, -(CH_2_)_3_, and -OH groups.
