# Supplementary material for: Improving Structural Homogeneity, Hydraulic Permeability, and Mechanical Performance of Asymmetric Monophasic Cellulose Acetate/Silica Membranes: Spinodal Decomposition Mix
Source: Membranes (Basel). 2023 Mar 17;13(3):346. doi: 10.3390/membranes13030346 (PMC10059883; doi:10.3390/membranes13030346)
Supplement: Supplementary file 1 [file membranes-13-00346-s001.zip › membranes-2197065-SI.pdf]

membranes MDPI

ANNEX I. Membrane Drying Process

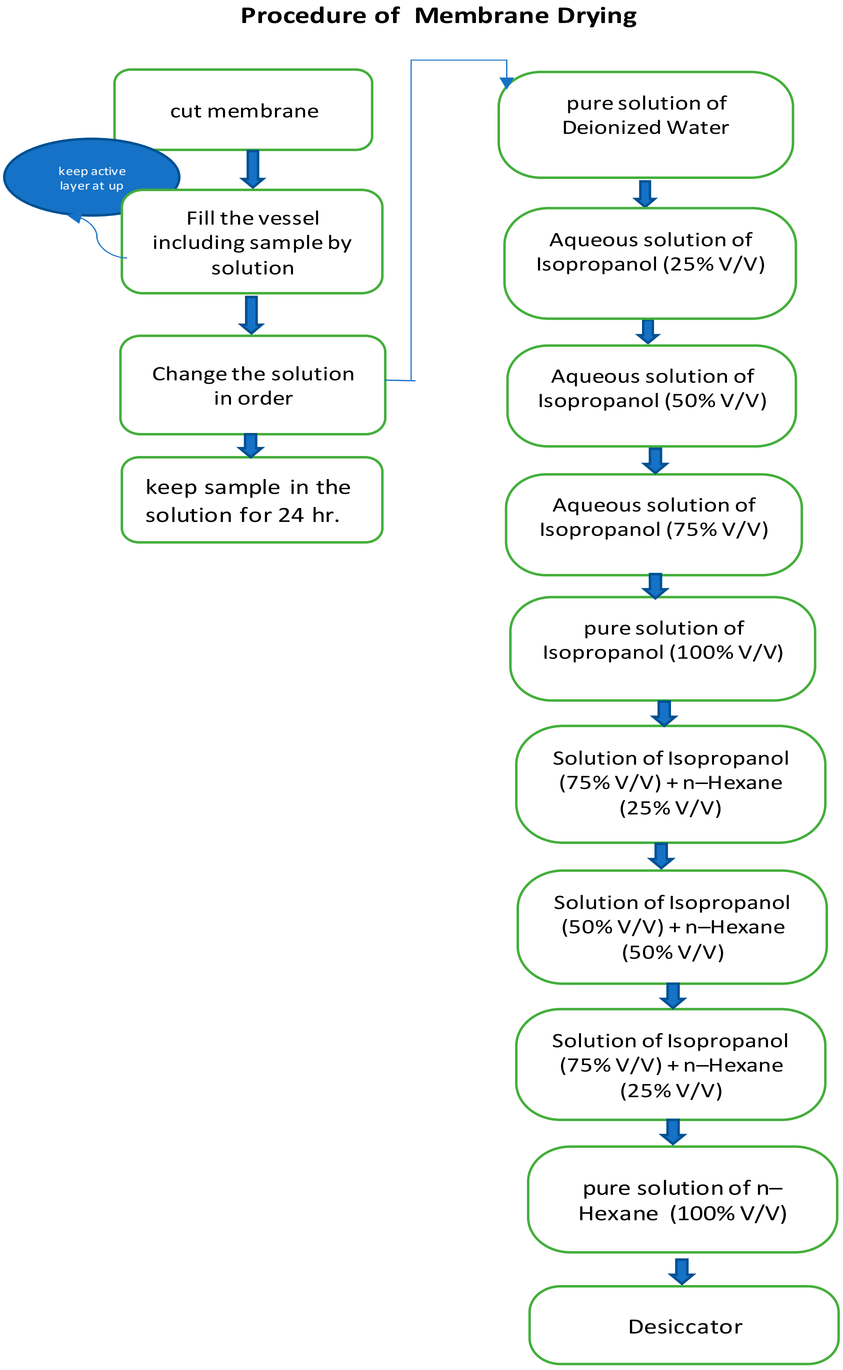

Figure S1. Flowchart of Membrane's Drying

Drying process efficiency was confirmed by FTIR-ATR analysis. In Figure S2 dry and wet membranes wide spectra are shown for both series. The drying process does not structurally damage any of the studied membranes, either in series1 or series2. The main differences are identified with the grey rectangle. The first rectangle with intense peaks for both wet membranes (serie1 and serie2) around 3386-3387cm-1 correspond to ns(OH) stretching vibration of molecular adsorbed water.

The second rectangle evidence the differences between the wet and dry membranes with an intense peak for wet membranes around 1635-1637 cm-1 that corresponds to the bending of the H-O-H bond from water.

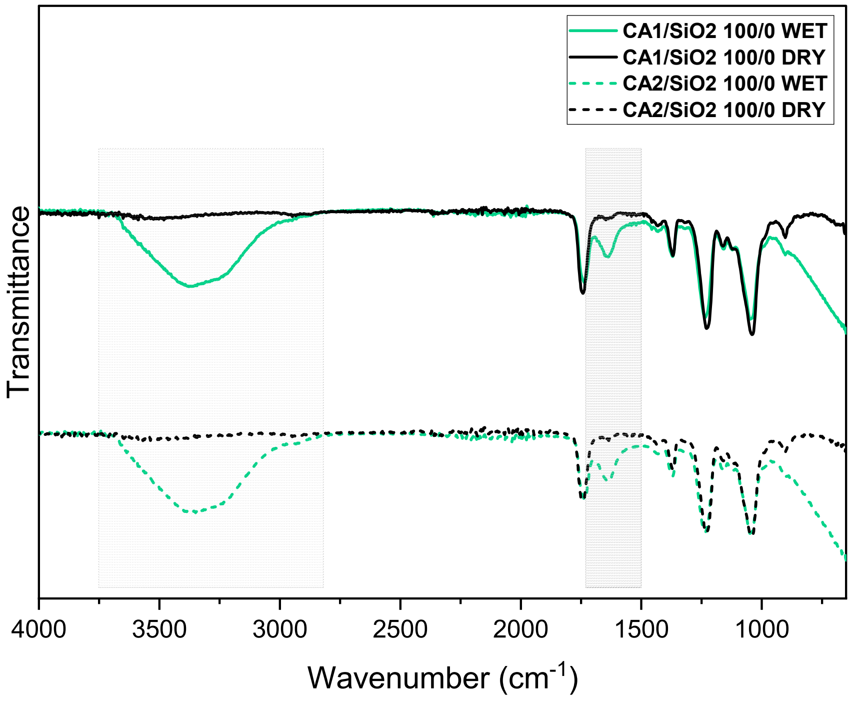

Figure. S2 – FTIR-ATR of pristine CA membranes (wet and dry).

ANNEX II Ultrafiltration experimental set-up and compaction optimization

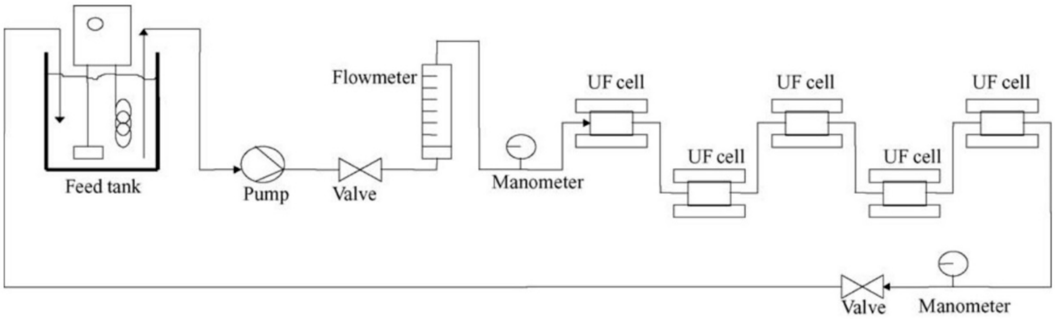

Figure **S3.** Ultrafiltration experimental set up.

Membrane's compaction reveals critical in hydraulic permeation reproducibility. Compaction optimization was experimentally determined until reaching a steady state regimen (Figure S4).

Compaction time vs. pure water flux was recorded for series1 and series 2 membranes. The flux was collected immediately after pressurization (and used as blank) and then collected hourly.

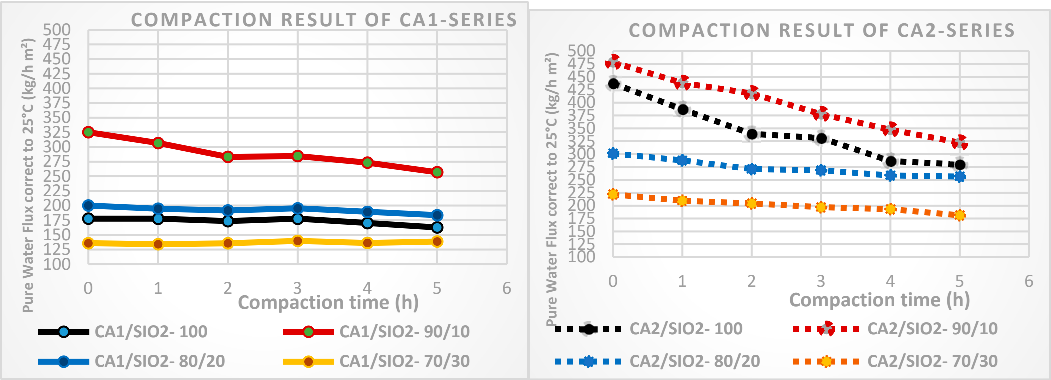

Figure S4. Compaction curves for series1 and series2.

LP Statistical Analysis:

Table. S1 – Significance values (p-value) adjusted by the Bonferroni correction for multiple tests between the different SiO_2 ratios of each series for the hydraulic permeability (Kruskal-Wallis test with pairwise comparisons for multiple tests).

	Series 1	Series 2
Pairwise Comparisons	Lp	Lp
100/0 - 90/10	1.000	1.000
100/0 - 80/20	0.129	0.727
100/0 - 70/30	0.002*	0.014**
90/10 - 80/20	0.523	0.727
90/10 - 70/30	0.014**	0.014**
80/20 - 70/30	1.000	0.807

Table. S2 – Significance (p-value) and U-test values between series for a fixed SiO_2 ratio for the hydraulic permeability (Mann-Whitney U Test).

Series 1 vs. Series2	Lp	
	p-value	*U*-value
100/0	0.032**	23.000
90/10	0.310	18.000
80/20	0.032**	23.000
70/30	0.032*	23.000

ANNEX III Mechanical Properties

Mechanical specimens were prepared with a "dog bone" shape to prevent the mechanical failure at the grips. A 3D printed cutting cast was design and produced to reduce the size variability and to minimize micro-fractures during the specimens' preparation. The cast was design with a 1 mm slit to allow for the constrained cut of the specimens with a sharp scalpel. The cross-sectional area of each specimen was assumed to be rectangular with a width equal to the distance between the slits of the cutting cast and a thickness equal to the thickness of the sheet to which it belonged. Moreover, the thickness of each sheet was also assumed to be uniform in the testing area. Its determination was performed by calculating the average value of the thickness measured at five randomly selected points. For this purpose, a mechanical micrometer with a precision of 0.01 mm (Mauser, Isny im Allgäu, Germany) was used. To ensure the same measuring conditions across all sheets, the micrometer was slowly closed until it detects resistance and blocks the mechanism. It is important to note that to avoid damaging the membranes during this procedure the points were selected not from the region of the gauge section, but from its vicinity.

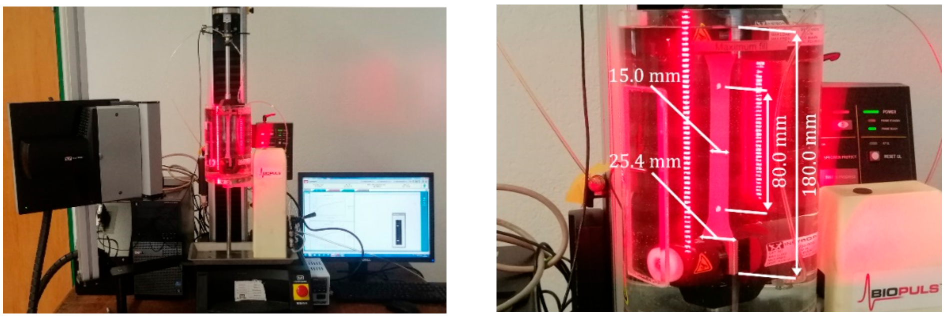

Figure. S5. a) Experimental setup used for the acquisition of the mechanical properties of the series1 and series2 membranes b) Representation of the specimen dimensions used during the tensile tests

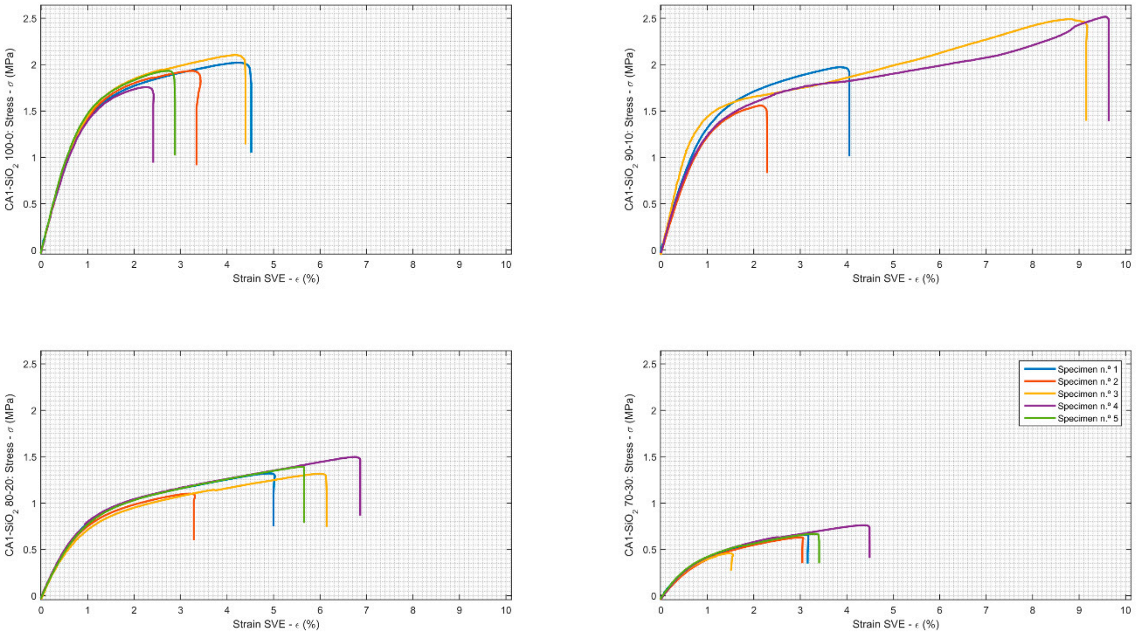

Figure. S6 – Stress-strain curves for the series CA1 with a SiO_2 composition of: a) 100% (top left); b) 90% (top right); c) 80% (bottom left); d) 700% (bottom right)

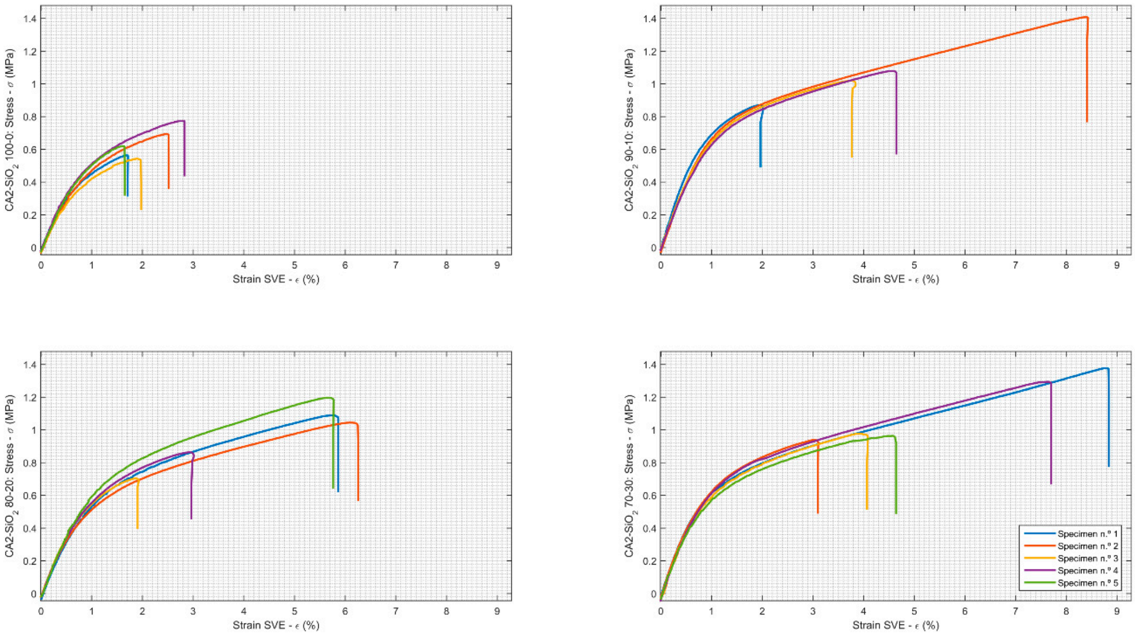

Figure. S7 – Stress-strain curves for the series CA2 with a SiO_2 composition of: a) 100% (top left); b) 90% (top right); c) 80% (bottom left); d) 700% (bottom right)

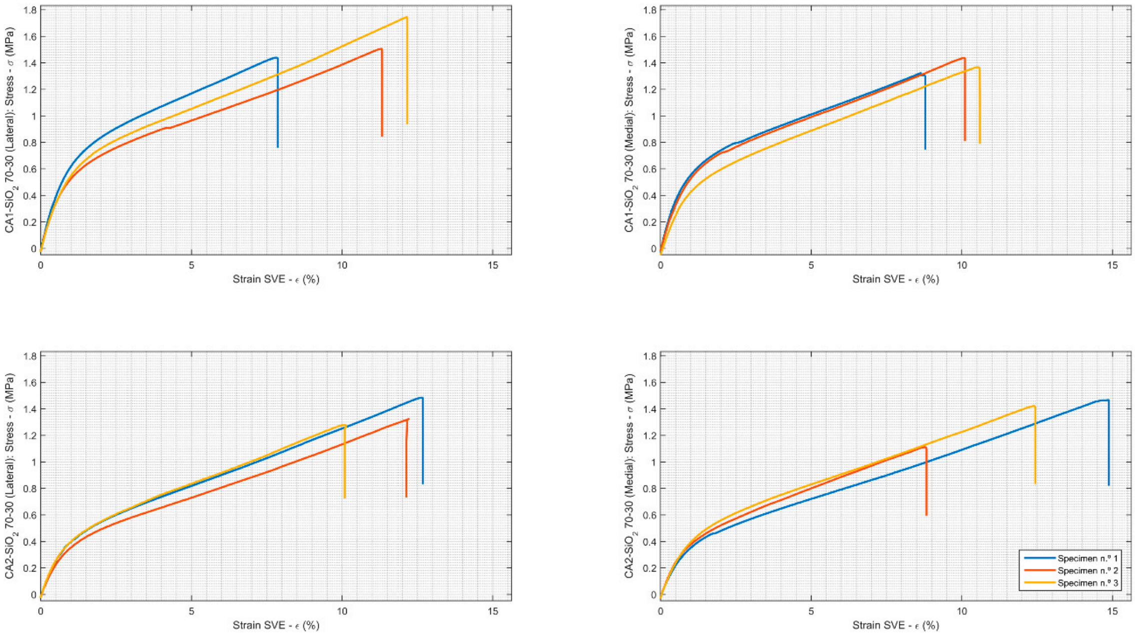

Figure. S8. Stress-strain curves for the series CA1 (top) and CA2 (bottom) with a SiO_2 composition of 70% (retest trials). Left charts represent the stress-strain relationship for specimens cut from the lateral side of the membrane sheets and the right ones from the medial part.

Table. S3. Significance values (p-value) adjusted by the Bonferroni correction for multiple tests between the different SiO_2 ratios of each series for the young's modulus, yield stress and yield strain parameters (Kruskal-Wallis test with pairwise comparisons for multiple tests).

	Series 1			Series 2		
Pairwise Comparisons	Young's Modulus	Yield Stress	Yield Strain	Young's Modulus	Yield Stress	Yield Strain
100/0 - 90/10	1.000	1.000	1.000	0.007*	0.027**	0.421
100/0 - 80/20	0.148	0.148	1.000	1.000	1.000	1.000
100/0 - 70/30	0.002*	0.002*	0.080***	0.008*	0.076***	0.353
90/10 - 80/20	0.766	0.766	1.000	0.219	0.131	0.187
90/10 - 70/30	0.026**	0.026**	0.025**	1.000	1.000	1.000
80/20 - 70/30	0.960	0.960	0.110	0.288	0.326	0.149

Table. S4. Significance (p-value) and U-test values between series for a fixed SiO_2 ratio for the young's modulus, yield stress and yield strain parameters (Mann-Whitney U Test).

Series 1 vs. Series2	Young's Modulus		Yield Stress		Yield Strain	
	p-value	U-value	p-value	U-value	p-value	U-value
100/0	0.004*	0.000	0.004*	0.000	0.004*	0.000
90/10	0.029**	0.000	0.029**	0.000	0.200	3.000
80/20	0.008*	0.000	0.008*	0.000	0.095***	4.000
70/30	0.008*	25.000	0.008*	25.000	0.008*	25.000

ANNEX IV. SEM image analysis

Table S5. Summary of images used to study the total thickness of series-1 membranes, and associated data.

Image name	Acronym/Composition	Magnification	FEGSEM image scale know distance	ImageJ conversion
F1-100-3cs2.tiff	CA1-SiO_2-100/0	2500x	50 µm	371600 pixels/µm
F1-90-2cs2.tiff	CA1-SiO_2-90/10	2500x	50 µm	371600 pixels/µm
F1-80-2cs2.tiff	CA1-SiO_2-80/20	2500x	50 µm	371600 pixels/µm
F1-70-3cs2.tiff	CA1-SiO_2-70/30	2500x	50 µm	371600 pixels/µm

Table S6. Summary of images used to study the total thickness of series 2 membranes, and associated data.

Image name	Acronym/Composition	Magnification	FEGSEM image scale know distance	ImageJ conversion
F2-100-2cs3.tiff	CA2-SiO_2-100/0	2500x	50 µm	371600 pixels/µm
F2-90-1cs2.tiff	CA2-SiO_2-90/10	2500x	50 µm	371600 pixels/µm
F2-80-2cs4.tiff	CA2-SiO_2-80/20	2500x	50 µm	371600 pixels/µm
F2-70-3cs2.tiff	CA2-SiO_2-70/30	2500x	50 µm	371600 pixels/µm

ANNEX V. IR assignments

Table S7. Water IR assignments in CA membranes

Wavenumber (cm^{-1})	Vibration	Structural unit	Reference
794<br>790 - 800	υ_S(Si-O)	≡Si-O-Si≡	[1–3]
980<br>965-995	υ_b(Si-O)	≡Si-OH	[1–3]
1046<br>1040 - 1050	υ(C-O)	-C-O-C-	[4,5]
1070	υ(C-O-C)	-C-O-C-	[1]
1070<br>1055-1090	ν_a(Si-O-Si)<br>(TO mode)	≡Si-O-Si≡	[1,3,6–8]
1160<br>1150-1165	ν_a(Si-O-Si)<br>(LO mode)	≡Si-O-Si≡	[1,3,6–8]
1123<br>1115 - 1175	ν(Si-O-C),	≡Si-O-R	[3,6–8]
1238<br>1228 - 1238	υ(C-O)	-C-O-C-	[3,5]
1430	δ(C-H)<br>(O-H)<br>adsorbed	-CH<br>-OH	[3,5,9]
1655<br>1650-1660<br>1640-1653	υ(C=O) free<br><br>δ(H-O-H)	HO-CH=O<br><br>H-O-H	[1,3,5,6]
1745	C<br>υ(C=O)<br>hydrogen<br>bonded	HO-CH=O	[3,6]
3000-3700	υ(O-H)	H-O-H<br>≡Si-OH<br>≡C-OH<br>unacetylated OH<br>groups (of the CA<br>polymer 3500 cm^{-1})	[3]
3755	ν_{as}(O-H)<br>vapor	H-O-H	[5]
3657	ν_s(O-H)<br>vapor	H-O-H	[5]
3604	$^{+}\upsilon$(O-H)	H-O-H	[9]
3472	O-H and<br>≡SiO-H	H-O-H···H_2O<br>≡SiO-H···H_2O	[1]

3408	++ υ(O-H)	H-O-H	[9]
3300-3400	ν(O-H) liquid	H-O-H	[5]
3200-3300	ν(O-H) ice-[illegible]	H-O-H	[5]

Table S8. Chemical properties of -COOH, -(CH2)3, and -OH groups

Functional Group	Chemical Formula	Structural Formula	Bond angles	Properties
carboxyl	-COOH	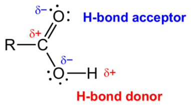	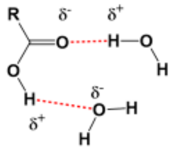	• polar<br>• charged<br>- R-COOH → R-COO⁻+H⁺<br>(acid character, ionize to release H⁺)<br>(weak acids)<br>• H-bonding (hydrophilic)
hydroxyl	-OH	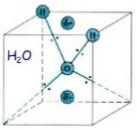	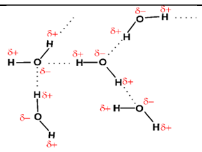	• polar<br>• charged<br>-OH + e⁻→ OH⁻<br>(ionize to accept e⁻)<br>• H-bonding (hydrophilic)
propyl	-CH2CH2CH3	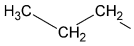		• apolar<br>• non- charged<br>• hydrophobic
formamide				• apolar<br>• non- charged<br>• hydrophobic
acetone				• apolar<br>• non- charged<br>• hydrophobic

References:

1. Al-Oweini, R.; El-Rassy, H. Synthesis and Characterization by FTIR Spectroscopy of Silica Aerogels Prepared Using Several Si (OR) 4 and R″ Si (OR′) 3 Precursors. *J Mol Struct* **2009**, *919*, 140–145.
2. Warring, S.L.; Beattie, D.A.; McQuillan, A.J. Surficial Siloxane-to-Silanol Interconversion during Room-Temperature Hydration/Dehydration of Amorphous Silica Films Observed by ATR-IR and TIR-Raman Spectroscopy. *Langmuir* **2016**, *32*, 1568–1576.
3. Minhas, F.T.; Farrukh, S.; Hussain, A.; Mujahid, M. Comparison of Silica and Novel Functionalized Silica-Based Cellulose Acetate Hybrid Membranes in Gas Permeation Study. *Journal of Polymer Research* **2015**, *22*, 1–13.
4. Murphy, D.; de Pinho, M.N. An ATR-FTIR Study of Water in Cellulose Acetate Membranes Prepared by Phase Inversion. *J Memb Sci* **1995**, *106*, 245–257.
5. Naghsh, M.; Sadeghi, M.; Moheb, A.; Chenar, M.P.; Mohagheghian, M. Separation of Ethylene/Ethane and Propylene/Propane by Cellulose Acetate–Silica Nanocomposite Membranes. *J Memb Sci* **2012**, *423*, 97–106.

6. Wojciechowska, P.; Foltynowicz, Z.; Nowicki, M. Celluloseacetate Butyrate Nanocomposites Synthesized via Sol-Gel Method. *Polimery* **2013**, *58*, 543–549.
7. Wojciechowska, P.; Foltynowicz, Z.; Nowicki, M. Synthesis and Characterization of Modified Cellulose Acetate Propionate Nanocomposites via Sol-Gel Process. *Journal of Spectroscopy* **2013**, *2013*.
8. Toprak, C.; Agar, J.N.; Falk, M. State of Water in Cellulose Acetate Membranes. *Journal of the Chemical Society, Faraday Transactions 1: Physical Chemistry in Condensed Phases* **1979**, *75*, 803–815.
